# Screening and Surveillance of Colorectal Cancer: A Review of the Literature

**DOI:** 10.3390/cancers16152746

**Published:** 2024-08-01

**Authors:** Marcello Maida, Dushyant Singh Dahiya, Yash R. Shah, Angad Tiwari, Harishankar Gopakumar, Ishaan Vohra, Aqsa Khan, Fouad Jaber, Daryl Ramai, Antonio Facciorusso

**Affiliations:** 1 Department of Medicine and Surgery, University of Enna ‘Kore’, 94100 Enna, Italy; marcello.maida@unikore.it; 2 Division of Gastroenterology, Hepatology and Motility, The University of Kansas School of Medicine, Kansas City, KS 66160, USA; 3 Department of Internal Medicine, Trinity Health Oakland/Wayne State University, Pontiac, MI 48341, USA; 4 Department of Internal Medicine, Maharani Laxmi Bai Medical College, Jhansi 284001, India; angadtiwari49@gmail.com; 5 Division of Gastroenterology and Hepatology, University of Illinois College of Medicine at Peoria, Peoria, IL 61605, USA; hgopakumarmd@gmail.com (H.G.); ishvohra28@gmail.com (I.V.); 6 Department of Internal Medicine, Parkview Health, Fort Wayne, IN 46805, USA; aqsa.khan@parkview.com; 7 Department of Internal Medicine, University of Missouri-Kansas City, Kansas City, KS 64110, USA; fouad.jaber@umkc.edu; 8 Division of Gastroenterology and Hepatology, The University of Utah School of Medicine, Salt Lake City, UT 84132, USA; darylramai@gmail.com; 9 Gastroenterology Unit, Department of Biomedical Science, Foggia University Hospital, 71122 Foggia, Italy

**Keywords:** colon cancer, screening, colonoscopy, surveillance

## Abstract

**Simple Summary:**

Colorectal cancer (CRC) is a significant health issue, being the third most common cancer and the second leading cause of cancer deaths worldwide. Most CRC cases are diagnosed at an advanced stage, especially in symptomatic patients, highlighting the need for effective early detection methods. While CRC is more prevalent in developed countries, its impact is felt globally and is influenced by factors such as diet, environment, and genetics. This study aims to review existing research on the effectiveness of various CRC screening methods. Early detection through screening significantly improves survival rates, making it crucial for better health outcomes. The review evaluates different screening tools and their cost-effectiveness and identifies populations that would benefit most from targeted screening. By improving screening strategies, the study aims to enhance early diagnosis and reduce CRC mortality rates, ultimately improving patient prognosis and quality of life.

**Abstract:**

Colorectal cancer (CRC) has the highest mortality rate among men and is the second highest among women under fifty, with incidence and mortality rates rising in younger populations. Studies indicate that up to one-third of patients diagnosed before fifty have a family history or genetic factors, highlighting the need for earlier screening. Contrariwise, diagnosis in healthy subjects through screening strategies enables early-stage detection of the tumor and better clinical outcomes. In recent years, mortality rates of CRC in Western countries have been on a steady decline, which is largely attributed to widespread screening programs and advancements in treatment modalities. Indeed, early detection through screening significantly improves prognosis, with stark differences in survival rates between localized and metastatic disease. This article aims to provide a comprehensive review of the existing literature, delving into the performance and efficacy of various CRC screening strategies. It navigates through available screening tools, evaluating their efficacy and cost-effectiveness. The discussion extends to delineating target populations for screening, emphasizing the importance of tailored approaches for individuals at heightened risk.

## 1. Introduction

Colorectal cancer (CRC) has become the most common cause of cancer-related deaths among men and the second most common among women under the age of 50 [1]. By 2030, CRC was predicted to rank second among women under 50 and first among males in terms of cancer-related fatalities. As CRC is largely curable when detected early through regular screening, this disturbing change emphasizes the essential need for early diagnosis. Interestingly, up to one-third of people diagnosed before the age of 50 have a family history or genetic vulnerability; this underscores the need to begin screenings earlier than the generally recommended age of 45 [1]. It is predicted that 152,810 Americans will be diagnosed with CRC this year, with 46,220 cases being rectal cancer and 106,590 cases being colon cancer. Overall, 53,010 people will unfortunately die as a result of these cancers [1]. The anticipated death rate from CRC has gone up as compared to the data of 2023 [1]. A sickness that is both detectable and curable should not claim a person’s life. Misclassification problems, however, occur when death certificates frequently report rectal cancer fatalities as colon cancer because of cultural and historical reluctance to use the term “rectum” [1]. The prevention of both colon and rectal cancers, as well as more precisely focused therapies, depend on accurate terminology and public knowledge. By addressing cultural stigmas, lives can be saved, and early detection improved.

Incidence rates of CRC have decreased since the mid-1980s as a result of evolving risk factors and increased screening among adults aged 50 and older. Overall rates fell by around 1% between 2011 and 2019; however, since the mid-1990s, rates for individuals under 55 have grown by 1% to 2% annually [1]. The mortality rate decreased by 56% from 29.2 per 100,000 in 1970 to 12.8 in 2021 as a result of improved therapies and early identification. However, the majority of these improvements are concentrated among older persons; since the mid-2000s, the death rate for those under 55 has increased by almost 1% each year [1]. In a study by O‘Connell et al. [2] (2003) examining data from 1973 to 1999, the incidence of colon cancer in individuals aged 20 to 40 years (*n* = 5383) increased by 17%, reaching a rate of 2.1 per 100,000 persons (*p* < 0.05). Additionally, the incidence of rectal cancer in this age group rose by 75%, with a rate of 1.4 per 100,000 persons (*p* < 0.05). According to Davis et al. [3] published in 2011, there was an increase in the incidence of CRC in young people below fifty years of age. Research work reviewed data from the Surveillance, Epidemiology, and End Results database from 1988 to 2006, and it demonstrated a 94% increase in rectal cancer diagnoses and a 67% increase in CRC cases in the 40–44 age group. Another study made a similar discovery, with the proportion of CRC amongst the population under 55 years increasing from 11% in 1995 to 20% in 2019, which also supports these results [4]. Despite significant advances in raising awareness of colorectal cancer screening, a recent study conducted by Dulskas et al. showed that, in Lithuania, only around 49.6% underwent screening over a 5-year period, with a CRC detection rate of 0.93–1.28% [5]. However, institutional parameters like coverage and uptake are still significant factors that impact effective CRC screening.

This literature review emphasizes how urgent it is to take preventative action against CRC, especially in younger people. Accurate nomenclature, more awareness, and early testing are crucial measures to address this emerging health concern and improve survival rates. On these premises, this review aims to revise the current evidence available in the literature to improve the performance and effectiveness of screening strategies for the early detection of CRC.

## 2. Screening and Surveillance for CRC

Only a small minority of CRC follows a hereditary pathway; most CRCs arise from adenomas through the so-called “adenoma-carcinoma sequence”, and the majority of adenomas present as polyps that progress from small to large, and then to dysplasia and cancer [6].

Neoplastic transformation is the result of interplay between both inherited and acquired genetic defects [7]. It has been postulated that this process takes at least 10 years on average [8]. Although nearly all CRCs arise from adenomas, only less than 5% of adenomas progress to cancer. Furthermore, some CRCs may arise from non-polypoid lesions, i.e., flat or depressed adenomas, a category accounting for about 30% of all adenomas [9]. Notably, large flat adenomas have a higher probability to harbor dysplastic changes or cancer than polypoid ones of similar size [10]. Anyway, the removal of adenomas prevents cancer: in patients undergoing endoscopic removal of one or more polyps, the incidence of colon cancer was 90% lower than in patients who had polyps that were not removed [11]. Polyp type also has an impact on the incidence of colorectal cancer. Patients with sessile serrate polyps (HR 1·74, 95% CI 1·08–2·79), tubulovillous adenomas (1·95, 1·69–2·24), and villous adenomas (3·45, 2·40–4·95) have a higher incidence of colorectal cancer compared to those with hyperplastic polyps (0·90, 0·76–1·06) or tubular adenomas (0·97, 0·84–1·12) [12]. Another relevant issue to underline is the increased rate of detection of right-sided/proximal colon cancers [13]. Even if such modification in the distribution of CRC may be partly due to increased sigmoidoscopy screening with the removal of adenomatous polyps in the left colon, a true raised incidence of right-sided CRCs has been demonstrated [14]. Colonoscopy screening had a higher benefit in terms of preventing colorectal cancer when compared to sigmoidoscopy, preventing 12 (95% CI, 10–14) colorectal cancer cases and four (95% CI, 3–5) colorectal cancer-related deaths as compared to sigmoidoscopy per 100,000 person-years [15].

## 3. Current Available Tools, Efficacy and Cost-Effectiveness

Screening for CRC enables the detection of neoplastic lesions at an early stage and also precancerous lesions, such as adenomas or sessile serrated lesions; this feature differentiates this screening program from others, such as prostate and breast cancers, which are only able to identify cancers at an early stage [16,17].

Currently, about 60–70% of CRCs are diagnosed at an advanced stage. If the healthy population was regularly submitted to annual screening, the death rate could be reduced to 60%, and 5-year survival increased to 73% (currently 65%) [4,18].

**Figure 1:**
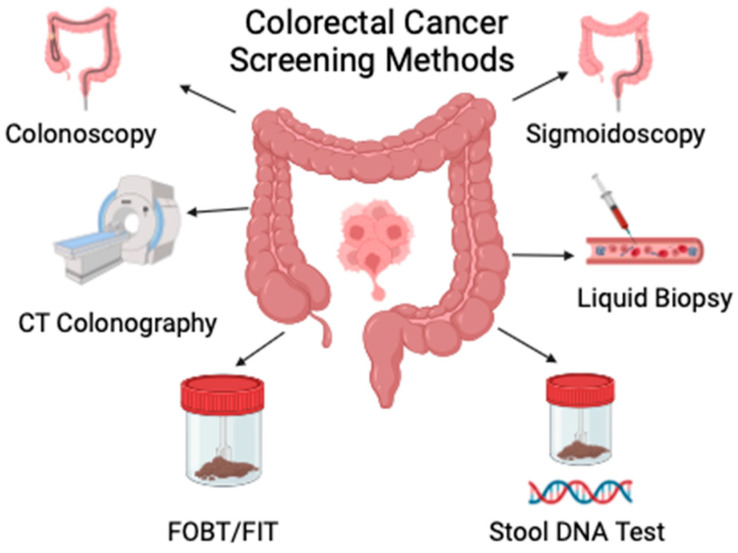
A graphical representation of commonly employed screening modalities for colorectal cancer (created using BioRender).

Guidelines on colorectal screening have been issued by several organizations, such as the American Cancer Society (ACS), the U.S. Preventive Services Task Force (USPSTF), the American College of Physicians (ACP), and the American College of Gastroenterology (ACG). There are several tests available for the screening of colorectal cancer, including colonoscopy, colon capsule, flexible sigmoidoscopy, computed tomographic colonography (CTC), and noninvasive stool tests, such as the guaiac fecal occult blood test (gFOBT), fecal immunochemical test (FIT), fecal DNA test, and the plasmic SEPT9 gene test [17]. A graphical representation of commonly employed modalities used for colorectal cancer screening is displayed in Figure 1.

### 3.1. Age-Appropriate Screening Guidelines

ACS 2018, USPSTF 2021, and ACG 2021 guidelines recommend screening for colorectal cancer from the age of 45 years for individuals with average risk; however, ACP 2023 guidelines recommend screening at the age of 50 years for individuals with average risk [19,20,21,22]. ACS 2018, USPSTF 2021, and ACG 2021 guidelines recommend screening for colorectal cancer for all individuals aged 50–75 years [19,20,22]. As per the ACS 2018, USPSTF 2021, and ACG 2021 guidelines, screening should be selectively considered in individuals between the ages of 75 and 85 years depending on the risk factors, and ACP 2023 guidelines recommend discontinuing screening if the life expectancy is 10 years or less [19,20,21,22].

### 3.2. Stool or Blood-Based Testing

#### 3.2.1. Guaiac Fecal Occult Blood Testing (gFOBT)

Guaiac FOBT (gFOBT) is among the first tests used in CRC screening. This test aims to identify the presence of hemoglobin in the feces by the action of peroxidase between the heme group and guaiac. It has a sensitivity of 12.9–79.4% and a specificity of 86.7–97.7% [23,24]. Another meta-analysis has reported a diagnostic accuracy of 25.5–86.3%, a sensitivity of 7.4–75%, and a specificity of 21.6–98.5% when using FOBT to screen for colorectal cancer [25]. Annual FOBT screening has reduced colorectal cancer mortality by 33% in 30 years; another study showed a reduction of 20% in 18 years [19]. The Nottingham trial, which included 152,850 individuals between the ages of 45 and 75 years, showed that there was a 13% reduction in CRC mortality (95% CI: 3–22%) in the intervention arm (biennial gFOBT test) [26]. There was an 18% reduction in colorectal cancer mortality when adjusted for non-compliance [26]. It is important to note that it can detect blood loss of >2 mL/day and can be affected by diets that include red meat, fruits, and vegetables containing vitamin C, as well as medications like non-steroidal anti-inflammatory drugs [27].

American Cancer Society (ACS) 2018 and USPSTF 2021 guidelines recommended annual testing with gFOBT for colon cancer screening. ACP 2023 guidelines recommend biennial testing with gFOBT for colon cancer screening. There are no specific recommendations regarding the interval of gFOBT testing for colon cancer screening in the ACG 2021 guidelines [19,20,21,22].

#### 3.2.2. Fecal Immunohistochemical Test (FIT)

FIT is currently the most widely used home-based stool-based screening test due to its higher sensitivity. It allows for the identification of bleeding from the lower gastrointestinal tract relating to many types of lesions, including precancerous ones, without requiring limitations on diet and requiring fewer stool samples than FOBT [28]. FIT has demonstrated sensitivity [79% (95% CI: 0.69 to 0.86)] and specificity [94% (95% CI: 0.92–0.95)] higher than the FOBT [29]. These data have been confirmed by studies that evaluated a total of 113,360 subjects, including 437 with a diagnosis of neoplasia at follow-up colonoscopy at 2 years. These studies also showed that repeating the test twice did not improve the detection capacity [28]. Annual screening with FIT over multiple rounds has been shown to reduce overall colorectal cancer detection by 80% [30]. Compared to FOBT, FIT has shown greater sensitivity in detecting precancerous lesions (20–50% vs. 11–20%, respectively) and colon cancer (20–50% vs. 79%, respectively), with greater compliance by patients [28,31] FIT can be qualitative or quantitative, with the first being more sensitive than the latter (0.82 vs. 0.73, χ^2^ = 3.933, *p* = 0.047) [32,33,34,35,36,37,38]. A 2022 Cochrane review of 33 articles showed that FIT has a higher sensitivity of 33% (95% CI: 27–40%; *p* < 0.001) compared to gFOBT, which has a sensitivity of 15% (95% CI: 12–20%) [39]. A positive qualitative FIT needs only the recognition of a colored band strip obtained using an immunoassay method; conversely, quantitative FIT requires specific tools, which are not always available, particularly in resource-limited settings. Therefore, the qualitative method is by far the most widely used in clinical practice [40].

ACS 2018, ACG 2021, and USPSTF 2021 guidelines recommended annual testing with FIT for colon cancer screening. ACP 2023 guidelines recommend biennial testing with FIT for colon cancer screening [19,20,21,22].

#### 3.2.3. Multi-Target Stool DNA Test

A fecal DNA test has recently been developed. The first major study was conducted by Imperiale et al. in 2014 on Cologuard and subsequently approved by the FDA in the same year [41]. This test can detect genetic alterations such as DNA mutations, microsatellite instability, altered DNA mismatch repair, and abnormal methylations in a stool sample [42]. It comprises an assay for mutant KRAS, methylated BMP3, methylated NDRG4, and a FIT for hemoglobin, which has a higher sensitivity for the detection of colorectal cancer (92 vs. 74%) but lower specificity (87 vs. 95%) compared to FOBT [19,24]. Sensitivity for mt-sDNA when detecting CRC was 92.3%, whereas the sensitivity of FIT was 73.8% (*p* = 0.002) based on a study using 9989 participants in which 65 (0.7%) had CRC and 757 (7.6%) had advanced precancerous lesions on colonoscopy [24]. Patients with positive mt-sDNA should undergo colonoscopy. A study of 12 patients conducted by Cooper et al. on patients with a negative colonoscopy after positive mt-sDNA from 11 to 29 months after colonoscopy showed that a repeat mt-sDNA test might identify polyps missed during colonoscopy, suggesting a high-quality colonoscopy with careful attention to the right colon should be performed [43].

mt-sDNA has a sensitivity of 90% for Stage I CRC, 100% for Stage II, and 92% for all stages, as compared to 66%, 76%, and 74%, respectively, for FIT. However, mt-sDNA had a higher false-positive rate, with a specificity of 86.6% for mt-sDNA compared to 94.9% for FIT [41]. The overall benefit of mt-sDNA every 3 years for colorectal cancer screening is questionable compared to annual FIT or colonoscopy every 10 years as it was found to be more expensive and less effective [44,45]. ACS 2018 and ACG 2021 guidelines recommend testing with mt-sDNA every 3 years for colon cancer screening [19,20]. USPSTF 2021 guidelines recommend testing with mt-sDNA every 1–3 years for colon cancer screening; however, ACP 2023 guidelines do not recommend mt-sDNA testing for colon cancer screening [21,22].

### 3.3. Serum Tests for Colorectal Cancer Screening

A liquid biopsy is a new technology used to detect tumor-related markers by analyzing circulating tumor cells, circulating tumor DNA, circulating free DNA or RNA, exosomes, circulating tumor-derived endothelial cells, or protein molecules [46]. A study to evaluate the ctDNA Lunar test to evaluate the performance of a cfDNA blood-based test on 7861 patients showed that it has a sensitivity of 83.1% (95% CI; 72.2–90.03) for the detection of colorectal cancer and a specificity of 89.6% (95% CI, 88.8–90.3) for any advanced neoplasia [47]. The plasma SEPT9 methylation assay was developed in 2008 by Lofton-Day [48]. Following this first test, marketed by Epigenomics AG as the EpiproColon 1.0, many companies have developed similar tests, even if they do not always yield the same results [49,50]. More recently, Epi proColon 2.0 has been made available, reporting good sensitivity (68.2%) and specificity (80%) in several case–control studies, cohort studies, and the PRESEPT study [51,52]. The Food and Drug Administration approved Epi proColon 2.0 in 2016; however, it is not recommended for colorectal cancer screening by the ACS 2018, USPSTF 2021, ACG 2021, and ACP 2023 guidelines [19,20,21,22].

### 3.4. Direct Visualization

#### 3.4.1. Colonoscopy

Colonoscopy is the only tool that allows for the evaluation of the entire colon and can also detect precancerous lesions and offer a means for their removal. It can be performed as a primary screening test or as a recall policy after a positive result of another noninvasive test [16,53,54,55,56,57]. Among screening tests, total colonoscopy seems to obtain the greatest reduction in mortality, with a value of approximately 68–88%, as proven by several case–control and randomized controlled studies. These data were also confirmed by a meta-analysis conducted by Brenner et al., which included six observational studies, highlighting how the mortality reduction was mainly due to the ability of colonoscopy to detect polyps and neoplasia of the proximal colon [57]. A similar study focusing on US veterans conducted by Kahi et al. showed a reduction in CRC mortality of 70% (odds ratio [OR]: 0.30; 95% CI: 0.24–0.38) for people undergoing colonoscopy [56]. However, the results from the NordICC Trial evaluating the benefit of screening of colonoscopy in 85,000 patients showed that there was a reduction in the risk of CRC over a 10-year period by 18% (risk ratio: 0.82; 95% CI: 0.70–0.93). However, there was no significant difference in the CRC-related deaths at 10 years in the invited group vs. the usual care group (0.28% vs. 0.31%) [58]. This study had several limitations, including the reduced number of participants that underwent colonoscopy in the intention-to-screen group (47%), as well as the low adenoma detection rate of 29% of endoscopists who participated in the study [58].

As the effectiveness of colonoscopy as a screening tool is dependent on the adequate detection and removal of colonic polyps, consistent quality measures are needed to help quantify healthcare processes and aid in providing high-quality healthcare [59]. The primary quality indicator for colonoscopy (depending on the performance of the endoscopist) is the adenoma detection rate (ADR). It is defined as the proportion of screening colonoscopies detecting at least one adenoma; ideally, it should be at least 25% [60]. Furthermore, the ADR is dependent on other quality measures, including cecal intubation rates, withdrawal times, and the quality of bowel preparation [59].

ACS 2018, USPSTF 2021, ACG 2021, and ACP 2023 guidelines unanimously recommend colonoscopy every 10 years for the screening of colon cancer [19,20,21,22].

#### 3.4.2. Flexible Sigmoidoscopy

Among endoscopic techniques, sigmoidoscopy has a better acceptability than colonoscopy. Flexible sigmoidoscopy allows for the visualization of the left side of the colon, removal of polyps, and referral for colonoscopy if at-risk lesions are found. At-risk distal lesions are defined as follows: multiple adenomas; at least one adenoma with high-grade dysplasia or a villous component >20%; and at least one polyp ≥ 10 mm in diameter [61]. The validity of sigmoidoscopy as a screening test for CRC has been supported by numerous randomized and controlled studies; in particular, the repetition of this examination at regular time intervals (3–5 years) seems to result in a reduced mortality rate of 26–31% for CRC when compared to non-intervention [51,62,63,64]. This reduction in mortality increases for tumors of the left colon, reaching a benefit of 46% (33–57%), as shown in a meta-analysis carried out by Brenner et al. that included 10 randomized controlled trials [64]. However, approximately 30% of these patients must then undergo colonoscopy for full evaluation.

The sensitivity and specificity of sigmoidoscopy, limited to the first 60 cm of the colon, are similar to those of colonoscopy, with a relatively lower risk of perforation [65]. Therefore, a choice concerning the characteristics of the ‘index’ polyp should be made given the cost/benefit ratio. Phase 3 studies showed that the RSS reduces mortality for CCR by 22–31% [51,62,66,67,68]. Furthermore, the SCORE study analyzed the implementation of sigmoidoscopy as a screening test for CRC with an 11-year follow-up; in this trial, the authors showed a reduction in mortality for CRC (hazard ratio [HR] = 0.70, 95% confidence interval [CI] = 0.54 to 0.91) with the use of RSS [69]. Another study, performed on a large cohort of asymptomatic patients aged 50 to 75 years, showed that sigmoidoscopy identified 70.3 percent of all subjects with advanced neoplasia and that a combination of one-time fecal occult blood test and sigmoidoscopy identified 75.8 percent of subjects with advanced lesions [70]. In the United States, the rates of flexible screening sigmoidoscopy have a downward due to the requirement for similar infrastructure that is used in colonoscopy and the need for colonoscopy in patients with adenoma on sigmoidoscopy; therefore, it is only recommended for patients who refuse FIT or colonoscopy [19].

ACS 2018 guidelines recommend flexible sigmoidoscopy every 5 years, whereas ACG 2021 guidelines recommend flexible sigmoidoscopy every 5–10 years [19,20]. USPSTF 2021 guidelines recommend flexible sigmoidoscopy every 5 years or every 10 years with annual FIT, and ACP 2023 guidelines recommend flexible sigmoidoscopy every 10 years with FIT every 2 years [21,22].

#### 3.4.3. Computed Tomography Colonography (CTC)

Computed tomographic colonography (CTC) can be a useful tool for patients who cannot tolerate colonoscopy and/or sedation. It carries a low radiation risk, although the cumulative risk of exposure to radiation with testing every 5 years actually remains unknown. It allows clinicians to evaluate 3D colonic images, recognizing polyps > 10 mm in 90% of cases and 6–9 mm polyps in 70–80% of cases [71,72,73]. A systematic review and meta-analysis of 49 studies comparing CTC with colonoscopy for screening conducted by Pickhardt et al. showed that the sensitivity of CTC is 96.1% (95% CI: 93.8–97.7%) compared to a sensitivity of 94.7% (95% CI: 90.4–97.2%) for colonoscopy; however, this study had a large amount of heterogeneity [74]. The inability to recognize small-sized polyps and flat adenomas (harboring greater malignant potential), representing more than 20% of colon cancer, as well as costs, are the limitations of this test [75,76]. Furthermore, to date, no RCTs have studied the impact of screening CTC on mortality reduction or the incidence of CRC [71,77,78]. Finally, the detection of extracolonic findings could lead to possible overdiagnosis and overtreatment of non-pathologic etiologies and may trigger panic, unnecessary investigations, and waste of healthcare resources [79,80,81].

ACS 2018, ACG 2021, and USPSTF 2021 guidelines recommend CTC every 5 years for the screening of colorectal cancer [19,20,22]. ACP 2023 guidelines do not recommend CTC for the screening of colorectal cancer [21].

#### 3.4.4. Capsule Endoscopy

Recent advancements in technology have led to the development of capsule endoscopy, which involves the use of a small pill that takes images while passing through the GI tract [82]. A systematic review of five studies evaluating the use of Pillcam Colon 2 showed a pooled sensitivity of 87% (95% CI: 77–93%) and a specificity of 76% (95% CI: 60–87%) for colorectal polyps ≥ 6 mm; however, 0.8% patients experienced capsule retention [82]. Another study focusing on 253 patients with positive immunohistochemical stain evaluated using capsule endoscopy followed by colonoscopy reported a sensitivity of 87% for capsule endoscopy (95% CI: 83–91%) and 88% for colonoscopy (95% CI: 84–92%) for polyps > 9 mm [83]. The TOPZ trial compared the outcomes of capsule endoscopy and CTC followed by colonoscopy in 286 patients and found the sensitivity and specificity for polyps ≥ 6 mm to be 79.2% and 96.3% for capsule endoscopy compared to 26.8% and 98.9% for CTC [84]. Capsule endoscopy was found to have higher sensitivity over CTC without a significant difference in specificity; however, none of the modalities are recommended for colorectal cancer screening by most organizations. ACG 2021 guidelines recommend capsule endoscopy every 5 years for colorectal cancer screening [19]. ACS 2018, USPSTF 2021, and ACP 2023 guidelines do not recommend capsule endoscopy for colorectal cancer screening [20,21,22].

In a recent microsimulation modeling study performed on a population undergoing CRC screening assuming 100% adherence, the strategies of colonoscopy every 10 years, annual FIT, sigmoidoscopy every 10 years with annual FIT, and CTC every 5 years performed from ages 50 through 75 years provided similar life years gained and a comparable balance of benefit and screening burden [16,71]. Therefore, the careful evaluation of the patient’s clinical status, comorbidities, and the risk–benefit ratio is always needed to ensure favorable clinical outcomes.

Table 1 summarizes the recommendations from various societies for colorectal cancer screening [19,20,21,22,85,86,87]. Table 2 summarizes the advantages and disadvantages of different screening modalities for colon cancer screening.

## 4. Recent Advances in Colonoscopy

EndoRings, a novel mechanical device manufactured by EndoAid Ltd. in Caesarea, Israel, promises to improve colonoscopy procedures. The flexible circular rings on this silicone rubber device, which fasten to the distal end of the colonoscope, enable the mechanical stretching of the colonic folds during withdrawal, stabilizing the scope tip centrally inside the lumen. Recent findings from a multicenter randomized study show that EndoRings considerably lower the miss rates for polyps (9.1% against 52.8% in a regular colonoscopy; *p* < 0.001) and adenomas (10.4% versus 48.3% in a normal colonoscopy; *p* < 0.001). According to these results, colonoscopy utilizing EndoRings may be more effective in detecting cancer than other techniques [88].

Endocuff (Arc Medical, Leeds, UK) is a mechanical device with projections that resemble fingers that are connected to the distal end of the endoscope. These projections flatten the colonic folds and center the scope tip during insertion and subsequently flare out after withdrawal. Endocuff-assisted colonoscopy considerably improves detection rates, as demonstrated by two randomized controlled studies, which found an 83% increase in adenoma detection and a 63% rise in polyp identification. Furthermore, compared to routine colonoscopy, the adenoma detection rate (ADR) increased from 21% to 35% (*p* < 0.0001), indicating that it is as effective as EndoRings in improving the results of colonoscopy [88].

Although there was a significant difference in the detection rates of adenomas smaller than 6 mm (443 vs. 378; *p* = 0.03) and flat polyps (213 vs. 161; *p* = 0.03) between Endocuff-assisted and standard colonoscopy, a subsequent randomized controlled trial with a larger patient cohort found no significant difference in the overall adenoma detection rate (ADR). Due to the lack of significant adverse events recorded, both EndoRings and Endocuff have shown excellent safety profiles. Mucosal lacerations are a modest danger, and there is a chance that the instrument will come loose from the colonoscope [88]. A meta-analysis of studies or larger randomized controlled trials evaluating the safety and efficacy of these newer devices will be helpful when making sound clinical decisions to improve ADR and the outcomes of colonoscopy.

Colonoscopy has recently benefited from a mechanical breakthrough called G-EYE (Smart Medical Systems Ltd.). A permanently connected balloon at the tip of the G-EYE endoscope is partially inflated during withdrawal to center the tip, straighten the colonic folds, and enhance endoscopic vision. In randomized tandem research, G-EYE colonoscopy was shown to have no major adverse effects and to have enhanced the adenoma detection rate (ADR) by 81% (*p* < 0.001) and decreased the adenoma miss rate (7.5% vs. 44.7%; *p* = 0.0002) when compared to traditional colonoscopy [89].

## 5. Artificial Intelligence in Colonoscopy

The development of artificial intelligence (AI) in gastrointestinal endoscopy has enabled colonoscopists to improve adenoma detection with the help of computer-aided detection (CADe), thereby decreasing the miss rate of clinically relevant lesions by 15–20% [90]. Various artificial intelligence systems, including GI Genius (Medtronic, Dublin, Ireland), EndoBRAIN-EYE (Cybernet Systems Corporation, Tokyo, Japan), ENDO-AID (Olympus Corporation, Tokyo, Japan), and EndoScreener (Wision A.I., Shanghai, China), are commercially available to facilitate adenoma detection during colonoscopy [91]. A meta-analysis of 21 studies comparing the benefits and risks of colonoscopy and CADe showed that ADR was significantly higher in the CADe group than in the standard group (4082 of 9090 [44.0%] vs. 3454 of 9142 [35.9%]; RR, 1.24 [95% CI, 1.16 to 1.33]. A lower adenoma miss rate was also detected in patients undergoing first colonoscopy with CADe as compared to those with standard colonoscopy with a risk ratio of 0.45 (CI, 0.35 to 0.58) [90]. However, the mean number of polypectomies for nonneoplastic lesions per colonoscopy was significantly higher in the CADe group as compared to the control group, with a mean difference of 0183 polypectomy ([CI, 0.107 to 0.258 polypectomy]; *p* < 0.001) [90]. Multiple studies have shown that CADx has a sensitivity ranging from 80.0 to 98%, a specificity ranging from 79.4 to 95.3%, an accuracy of 74.4–94.0%, and negative predictive values ranging from 73.5 to 97% for the histological prediction of colorectal polyps [92]. In the future, new technological developments are expected to improve the diagnostic sensitivity of colonoscopy in detecting adenomas, reducing the miss rate of advanced lesions.

The introduction of CADe-assisted colonoscopy will initially increase costs since more adenomas are detected, resulting in more pathological analyses and shorter surveillance times. Nevertheless, these costs may be mitigated by other benefits, including long-term monetary savings and decreased incidences of colorectal cancer. Mori et al. (2022) [93] found that in a CADe group, the percentage of such recommendations increased from 8.4% in the control group to 11.3% in the latter. This shows that a 35% relative increase in intense surveillance recommendations is linked to CADe technology. This implies that CADe helps identify more patients who could benefit from closer monitoring, potentially leading to better outcomes in terms of early diagnosis and treatment. On the other hand, Areia et al. [94] also revealed that, in the United States, the use of CADe could reduce 7194 new cases of colorectal cancer in addition to 2089 deaths every year, with cost savings of USD 290 million. The World Endoscopy Organization has stressed that while CADe increases the initial cost, it may reduce the general cost of handling cancers in the future [95]. Moreover, AI in colonoscopy can increase the uniformity of global care quality and equality of healthcare access since machine learning consultation is available to everyone all over the world.

## 6. Screening in Individuals with Baseline Risk of CRC

Several factors are known to increase the risk of CRC in the general population. In this section, we discuss the factors that influence the recommendations for screening and surveilling CRC.

### 6.1. Family History

One out of four patients with CRC have a positive family history of colorectal cancer [8]. Hence, clinicians must investigate the family history of CRC, focusing on the presence of a previous diagnosis of CRC or adenomatous polyps among family members, and if found, the age of the family member at the time of diagnosis, the number of polyps/cancers found, and the degree of kinship (first-degree versus second-degree relatives) [96]. CRC may also occur earlier in life, even between 30 and 40 years of age, in patients with a family history of CRC [97]. Interestingly, patients with a family member with a history of adenomatous polyp ≥ 1 cm, with high-grade dysplasia, or with villous elements may also be at increased risk of CRC [98]. As a result, several guidelines have similar recommendations for CRC screening in patients with a family history [23,99,100,101].

If a single first-degree relative was diagnosed with CRC or an advanced adenoma at the age of 60 years or older (≥1 cm, or with high-grade dysplasia and/or villous elements), screening with colonoscopy is recommended at age 40 years and is to be repeated every 10 years [19]. However, if a single first-degree relative was diagnosed with CRC or an advanced adenoma before the age of 60 years, or two or more first-degree relatives had CRC or advanced adenomas at any age, screening with colonoscopy is recommended at the age of 40 or 10 years before the youngest family member’s diagnosis, and colonoscopy is to be repeated every 5 years [19].

### 6.2. Familial Adenomatous Polyposis (FAP)

FAP is an autosomal-dominant disease caused by mutations in the adenomatous polyposis coli gene [102]. Beginning in adolescence, hundreds to thousands of polyps develop throughout the colon, and nearly all patients ultimately develop CRC. Of note, patients with FAP are also at risk of several extracolonic malignancies. Consequently, genetic tests are recommended for at-risk family members of known FAP carriers or in individuals with more than 100 adenomas [102]. In gene carriers, annual colonoscopy starting at the age of 10 to 15 years is recommended [103]. In patients with numerous polyps, multiple large (>1 cm) adenomas, or advanced adenomas, colectomy should be performed. Furthermore, patients who have undergone proctocolectomy require pouchoscopy for cancer surveillance [86,104].

An attenuated form of FAP is also caused by APC mutations. It is characterized by fewer polyps, a later age of onset, and a lower risk of developing CRC [105]. In this context, genetic testing of at-risk family members of known attenuated FAP families or in individuals with more than 10–20 adenomas is recommended. Annual colonoscopy in gene carriers should be performed starting at the age of 20–25 years. Even if patients with attenuated FAP may be managed with polypectomy plus surveillance, prophylactic colectomy is recommended when adenomas are too numerous or difficult to manage with endoscopic polypectomy [106].

### 6.3. Hereditary Nonpolyposis Colorectal Cancer (HNPCC)

HNPCC, also known as Lynch syndrome, is an autosomal-dominant disorder with high penetrance. Accounting for approximately 3% of all colonic adenocarcinomas, the syndrome is caused by germline mutations in one of the several DNA mismatch repair genes [107]. The colon cancers seen in Lynch syndrome differ from sporadic CRC relating to two characteristics, i.e., they develop at an earlier age and mainly involve the right colon. In addition, approximately 10% of patients with Lynch syndrome have synchronous or metachronous cancers, and the mean age at the time of diagnosis of colon cancer is around 48 years, with some patients presenting as early as 20 years of age [108]. Similar to FAP, Lynch Syndrome is also associated with a high risk of several extracolonic tumors [109]. Hence, for patients with HNPCC, annual colonoscopy may be recommended starting at the age of 20–25 years or 10 years prior to the earliest age of colon cancer diagnosis in the family [110,111].

### 6.4. Colonic Inflammatory Bowel Disease

Patients with longstanding colonic inflammatory bowel disease (IBD) have an increased risk of CRC compared to the general population [112]. Of note, most available evidence on CRC in IBD patients is derived from studies in patients with ulcerative colitis (UC), while little is known about colonic Crohn’s disease (CD). Hence, most recommendations on screening primarily apply to patients with UC. The risk of CRC in UC is associated with disease duration and extent, i.e., cumulative CRC risks of 2% at 10 years, 8% at 20 years, and 18% at 30 years of disease duration have been reported, and patients with extensive colitis carry the highest risk; however, the risk of developing CRC is not increased in patients with disease limited to the rectum [113].

Given these data, an initial colonoscopy screening is highly recommended for all patients with ulcerative colitis, independent of the known extent of colitis, in order to re-evaluate the extent of the disease and rule out the presence of dysplastic lesions. The suggested timing for this endoscopic examination is 8 years after the beginning of symptoms [114,115,116]. In patients with concurrent primary sclerosing cholangitis, surveillance colonoscopies should be performed yearly as the risk of developing CRC in these patients is very high [115,116,117], may occur early in the course of the disease [118], and tumors frequently develop in the right colon [119]. Conversely, the risk of CRC is only minimally increased in patients with proctitis who have no other risk factors; therefore, no regular screening is required [120]. In both extensive and left-sided colitis, risk stratification depends on four predominant factors: the presence of pancolitis, history of pseudopolyps, persistent endoscopic and/or histological inflammation, and family history of CRC. Based on these factors, patients can be stratified as low-risk (colonoscopy every 3–4 years) or high-risk (colonoscopy every 1–2 years) [115,121]. Furthermore, in recent years, advanced endoscopic imaging (chromoendoscopy, electronic chromoendoscopy, image-enhanced endoscopy, confocal laser endomicroscopy, endocytoscopy, and fluorescence endoscopy) and techniques involving the detection of alterations in mucosal antigens and genetic abnormalities (sialosyl-Tn) have been investigated for the surveillance of patients with IBD. Thus far, studies have yielded promising results and may lead to more efficient surveillance, particularly in high-risk IBD patients [122,123].

### 6.5. Abdominal Radiation

Patients who received abdominal radiation due to childhood malignancies have an 11-fold increased incidence of CRC compared to individuals not exposed to radiation during childhood [124,125]. The Children’s Oncology Group recommends a colonoscopy every five years for survivors of childhood cancer who received abdominal radiation, with screening beginning five years after radiation or at the age of 30 years [126,127].

## 7. Surveillance Strategies

After an index screening examination, the selected test should be repeated at specific intervals over time. The timing of surveillance typically differs depending on the primary test and test results.

Globally, fecal occult blood tests (FOBTs and FITs) are economic, noninvasive, well tolerated, and have broad applications. If the test result is negative, high-quality evidence supports new testing every year or every 2 years; however, in the case of a positive test result, colonoscopy has to be used as a recall strategy with the aim of confirming or excluding the presence of CRC [19,85].

Fecal tests with molecular markers in combination with FIT (FIT-DNA) have also been approved by the Food and Drug Administration (FDA) for CRC screening. While the screening interval for FIT is every 1 year, mt-sDNA should be checked every 3 years [19]. The best time-to-colonoscopy after a positive fecal test needs to be properly defined.

### 7.1. Surveillance after Endoscopy

A colonoscopy can be performed as the primary screening test or as a recall policy after a positive result of another primary screening test. On endoscopy, if no adenoma is detected, the second surveillance colonoscopy should be planned in 10 years, whereas if colonic adenomas are detected and removed, the timing of surveillance needs to be shortened. Although CTC and capsule endoscopy are not generally recommended, screening should be performed every 5 years if these modalities are used for initial screening [19].

Based on the type of polyp removed, i.e., serrated polyps (which can be neoplastic or non-neoplastic) or adenomatous polyps, surveillance intervals may differ as follows.

#### 7.1.1. Serrated Polyps

Serrated polyps are a heterogeneous group of lesions with variable malignant potential. They include hyperplastic polyps, traditional serrated adenomas, and sessile serrated adenomas [128]. It is now known that there are several subtypes of serrated polyps, some of which can develop into invasive cancer by way of the serrated neoplasia pathway. The classification system used for serrated polyps is changing. Based on the presence or absence of dysplasia, these polyps are divided into two main types according to the most recent World Health Organization (WHO) classification. Sessile serrated adenomas/polyps (SSA/Ps), hyperplastic polyps, SSA/Ps with cytological dysplasia, and classic serrated adenomas (TSAs) are among the subtypes [129].

The risk of neoplasia after finding a sessile polyp is low compared to that of tubular adenoma. Three-year surveillance colonoscopy is recommended for sessile serrated polyps of at least 10 mm in diameter or with dysplasia if there are 5–10 serrate polyps without high-risk features [130]. For sessile serrated polyps without high-risk features, a five- to ten-year follow-up colonoscopy is recommended for 1–2 polyps, and a three to five-year follow-up colonoscopy is recommended for 3–4 polyps [130]. A three-year follow-up colonoscopy is recommended for traditional serrated adenomas as they confer a higher neoplasia risk than tubular adenomas [130]. Hyperplastic polyps < 10 mm in diameter do not have an increased risk of neoplasia and can be monitored every 10 years. For three- to five-year follow-up, colonoscopy is recommended for a hyperplastic polyp > 10 mm, and individualized follow-up is recommended for >20 hyperplastic polyps suggestive of serrated polyposis syndrome [130].

#### 7.1.2. Adenomatous Polyps

About two-thirds of all colonic polyps are adenomas, which are, by definition, dysplastic and thus have malignant potential. Low- and high-grade dysplasia should only be recognized, and the terms “carcinoma in situ” or “intramucosal adenocarcinoma” should only be included in the condition of high-grade dysplasia [131]. Adenomas with high-grade dysplasia may also coexist with areas of invasive cancer in the polyp. Within an individual adenoma, three factors are associated with an increased risk of focal cancer: polyp size, villous histology, and high-grade dysplasia [131]. Adenomatous polyps > 1 cm in diameter are at risk for the development of CRC and metachronous cancer [132]. Of note, the proportion of adenomas with high-grade dysplasia or >25 percent villous histology increases from 1 to 2% in small adenomas (<5 mm) to 7–12% for medium-sized adenomas (5 to 10 mm) and 20–30% for adenomas >1 cm [133]. Consequently, villous histology (and dysplasia) is closely linked with the size of the polyp. On the basis of such evidence, and to simplify the recommendations for surveillance and management, adenomatous polyps are commonly divided into two main categories: low-risk and advanced adenomas [134].

#### 7.1.3. Low-Risk Adenomas

Those who have had a baseline colonoscopy and found to have one or two tubular adenomas less than ten millimeters in size should return to screening rather than undergoing colonoscopy surveillance, as per the most recent 2020 recommendations released by the European Society of Gastrointestinal Endoscopy (ESGE) [135]. Public Health England (PHE), the British Society of Gastroenterology (BSG), and the Association of Coloproctology of Great Britain and Ireland (ACPGBI) all urge that screening be resumed for those with 1–4 adenomas of less than 10 mm [136].

United States Multi-Society Task Force (USMSTF) guidelines recommend a seven-ten-year follow-up colonoscopy for 1–2 adenomas without high-risk features and a three- to five-year follow-up for 3–4 adenomas [136]. The ESGE guidelines urge colonoscopy surveillance every three years for those with five or more adenomas [135]. Similarly, the US Multi-Society Task Force (USMSTF) and Korean standards suggest a three-year monitoring interval for people with 5–10 tubular adenomas of less than 10 mm [136]. As such, the risk of colorectal cancer was found to be 74% lower among those who formerly tested negative on colonoscopy. Therefore, screening should be carried out routinely. The protective effect remained even when the colonoscopy was performed twenty years before. It is worth noting that rectum and sigmoid tumors showed a more conspicuous decrease in terms of risk. Moreover, people who have had multiple negative colonoscopies have much lower chances than those with only one negative result. Consequently, performing a colonoscopy once every ten years after a negative finding was cost-effective, as illustrated by these findings. These results help to maintain its reputation as an affordable screening method and also emphasize the long-term benefits it has in reducing the risk of colorectal cancer [137].

#### 7.1.4. Advanced Adenomas

Advanced adenomas are polyps of ≥10 mm with villous histology or high-grade dysplasia. If one or more advanced adenoma is detected at baseline colonoscopy, the first surveillance colonoscopy should be performed within 3 years. Therefore, the endoscopist has the main responsibility to establish the surveillance interval and provide a written recommendation, considering, together with the histological findings, the following four issues: (a) the completeness of the colonoscopy; (b) the withdrawal time; (c) the characterization of each lesion detected, according to the current classifications; and d) the endoscopic techniques used (i.e., “en bloc” polypectomy is more radical than “piecemeal” polypectomy), according to the size and the morphological features of the lesions [138].

## 8. Strategies for Clinical Practice

Colorectal cancer represents a significant public health concern that has high rates of morbidity and mortality. Due to the slow rate of transformation from premalignant lesions to carcinoma, the detection of early-stage disease through adequate screening tests can reduce the incidence and mortality of CRC as the removal of colonic polyps can prevent progression to cancer. Current evidence supports the overall effectiveness of screening for CRC on the basis of several high-quality studies performed on large populations.

While the effectiveness of screening has been clearly demonstrated, there is greater uncertainty concerning the best strategy to be applied for screening. To date, no screening strategy for CRC can be defined as universally ideal. Therefore, the best is that which is economically viable and which the patient can adhere to over time.

We suggest performing a colonoscopy every 10 years or, if not possible, biannual FIT for people with normal baseline risk. However, for individuals with high baseline risk or hereditary syndromes, colonoscopy should be performed with shortened surveillance intervals according to international guidelines. Computed tomographic colonography (CTC) represents a viable alternative to colonoscopy for patients who cannot tolerate colonoscopy. However, it is important to consider that its accuracy is low for the detection of small (<1 cm) and flat colonic polyps and that it exposes patients to ionizing radiation. Furthermore, independent of the index screening test, any positive result requires a colonoscopy as a recall strategy. As previously mentioned, colonoscopy is the only tool that allows for an evaluation of the entire colon, detects precancerous lesions, and also provides means for their removal. Additionally, just performing a colonoscopy is not enough. Consistent quality measures, which could help quantify the healthcare processes and aid in providing high-quality healthcare, are needed to preserve the effectiveness of CRC prevention and improve patient outcomes.

Overall, considerable strides have been made in CRC screening, resulting in increased survival rates. However, in the future, efforts must be made to optimize the quality of prevention. First, health policy interventions aimed to modify behavioral risk factors in asymptomatic people already at increased risk (primary prophylaxis) of CRC should be implemented. Second, it is mandatory to improve the quality of early detection of tumors (secondary prophylaxis) by refining the current knowledge of risk factors and improving the performance of screening tests. Further studies will be necessary in order to refine the individual risk profile for CRC. Several risk factors (e.g., comorbidities like diabetes, thyroid disease, and metabolic syndrome) currently under investigation should be better studied to identify possible further target populations that could enter a screening program for CRC [139,140,141,142]. Similarly, accurate genetic tests for the confirmation of hereditary syndromes will be needed to screen family members and assess their risk with a noninvasive method. This could be helpful in terms of tailoring screening strategies based on individual risk using a personalized approach, thereby increasing the accuracy of screening by minimizing unnecessary examinations and reducing associated risks. Furthermore, screening techniques, especially colonoscopy, need to be technologically improved in order to maximize their diagnostic sensitivity. With the recent development of serum-based markers for colorectal screening, it will also be useful to develop and explore other screening modalities that would involve salivary or urinary testing for the screening for colorectal cancer. Noninvasive testing with high sensitivity and specificity will likely contribute to increased adherence, which can be followed by endoscopic evaluations based on the results of initial screening tests.

Another debated topic is when to stop surveillance. As discussed above, screening for CRC should be started in all patients at risk and stopped as soon as it becomes reasonably futile, for instance, when the age or general status of the patient excludes every “a priori” treatment option for the tumor. International guidelines recommend beginning screening at 45 years of age in at-risk populations, tailoring screening for patients between 76 and 85 years of age on the basis of comorbidity, and stopping screening for patients aged more than 85 years [19,100,143]. It is also equally important to use sustainable approaches since endoscopy also has some adverse influences on the environment. Based on empirical evidence [144], studies have shown that practically every procedure carries a sizable carbon impact. Reusable medical equipment, encouraging teleconsultations, and optimizing procedures using noninvasive methods and updated recommendations are some strategies to lessen this burden.

## Figures and Tables

**Table 1:** Summary of recommendations from various societies for colorectal cancer screening.

	ACG 2021	USMSTF 2022	ACS 2018	USPSTF 2021	NCCN 2022	ACP 2023
Age	Begin avg risk at 45 years	Begin avg risk at 45 years	Begin avg risk at 45 years	Begin avg risk at 45 years	Begin avg risk at 45 years	Begin avg risk at 50 years
All	50–75 years	50–75 years	50–75 years	50–75 years	50–75 years	50–75 years
75–85 years	Selectively	Selectively	Selectively	Selectively	Selectively	Discontinue at 75 years if life expectancy is 10 years or less
85+ years	Discourage screening	-	Discourage screening	Discourage screening	Discourage screening	-
Stool Based						
gFOBT	-	-	Annual	Annual	Annual	Every 2 years
FIT	Annual	Annual	Annual	Annual	Annual	Every 2 years
mt-sDNA	Every 3 years	Every 3 years	Every 1–3 years	Every 1–3 years	Every 3 years	-
Endoscopic						
Flexible Sigmoidoscopy	Every 5–10 years	Every 5–10 years	Every 5 years or Every 10 years if combined with FIT	Every 5 years	Every 5 years or Every 10 years if combined with FIT	Every 10 years + FIT every 2 years
Colonoscopy	Every 10 years	Every 10 years	Every 10 years	Every 10 years	Every 10 years	Every 10 years
CT Colonography	Every 5 years	Every 5 years	Every 5 years	Every 5 years	Every 5 years	-
Colon Capsule	Every 5 years	If the patient refuses all of the above	-	-	-	-

ACG: American College of Gastroenterology; USMSTF: United State Multi-Society Task Force; ACS: American Cancer Society; USPSTF: United States Preventive Services Task Force; NCCN: National Comprehensive Cancer Network; ACP: American College of Physicians; gFOBT: guaiac fecal occult blood test; FIT: fecal immunohistochemical test; mt-sDNA: Multi-Target Stool DNA.

**Table 2:** Summary of advantages and disadvantages of different screening modalities for colon cancer screening.

Screening Method	Technique	Advantages	Disadvantages
Colonoscopy	Involves the insertion of a flexible tube with a camera (colonoscope) into the rectum to examine the entire colon for polyps or cancerous growths.	-Direct visualization allows for the detection and removal of precancerous polyps during the procedure.	-Requires bowel preparation, which may be uncomfortable. -Invasive procedure with a small risk of complications, such as bleeding or perforation.
Flexible Sigmoidoscopy	Involves the insertion of a thin, flexible tube with a camera (sigmoidoscope) into the rectum and lower part of the colon to examine for polyps or cancerous growths.	-Less invasive than colonoscopy. -Does not require full bowel preparation.	-Limited in scope compared to colonoscopy; only examines the lower part of the colon. -Polyps or cancers in the upper colon may be missed. -Positive findings require follow-up colonoscopy.
CT Colonography	Uses CT scans to create detailed images of the colon and rectum, allowing for the detection of polyps or cancerous growths.	-Noninvasive and does not require sedation. -No risk of perforation. -Provides detailed images of the entire colon.	-Requires bowel preparation similar to colonoscopy. -Polyps found may require follow-up colonoscopy for removal. -Radiation exposure from CT scans.
Capsule Endoscopy	Capsule endoscopy involves swallowing a small camera that captures images of the colon as it passes through the digestive tract.	-Noninvasive -No need for sedation -Provides comprehensive visualization of the entire GI tract -Better detection of polyps and early cancers	-Requires bowel preparation -Limited availability and accessibility -Risk of capsule retention -Not therapeutic; positive findings require follow-up colonoscopy -Possible technical issues (e.g., battery life, transmission problems)
Fecal Immunochemical Test (FIT)	A stool-based test that detects hidden blood in the stool, which can be a sign of colorectal cancer or polyps.	-Noninvasive and simple to perform at home. No dietary or medication restrictions before the test. -No need for bowel preparation.	-Can produce false-positive results due to bleeding from other sources (e.g., hemorrhoids). -Sensitivity may vary, and polyps or early-stage cancer may not always be detected. -A follow-up colonoscopy is required if the test is positive.
Stool DNA Test (mt-sDNA)	A stool-based test that combines FIT with analysis of DNA markers associated with colorectal cancer.	-Higher sensitivity for detecting advanced adenomas and colorectal cancer compared to FIT alone. -Noninvasive and can be performed at home without dietary or medication restrictions.	-More expensive than FIT. -False-positive results can occur, leading to unnecessary follow-up testing. -Requires collection of multiple stool samples.
gFOBT	gFOBT detects hidden blood in stool samples, suggesting bleeding from polyps or cancer using guaiac-based chemical tests.	-Noninvasive -Easy to perform at home -No need for bowel preparation -Low cost -No sedation needed	-Lower sensitivity and specificity compared to other methods -High false-positive rate -Requires multiple samples -Dietary restrictions prior to the test -Not diagnostic; positive results need follow-up colonoscopy -Can miss polyps and early cancers

ACG: American College of Gastroenterology; USMSTF: United State Multi-Society Task Force; ACS: American Cancer Society; USPSTF: United States Preventive Services Task Force; NCCN: National Comprehensive Cancer Network; ACP: American College of Physicians; gFOBT: guaiac fecal occult blood test; FIT: fecal immunohistochemical test; mt-sDNA: Multi-Target Stool DNA.
